# Microstructure Evolution of the Semi-Macro Segregation Induced Banded Structure in High Strength Oil Tubes During Quenching and Tempering Treatments

**DOI:** 10.3390/ma12203310

**Published:** 2019-10-11

**Authors:** Bo Li, Ming Luo, Zhanbing Yang, Feifei Yang, Huasong Liu, Haiyan Tang, Zhonghua Zhang, Jiaquan Zhang

**Affiliations:** 1 School of Metallurgical and Ecological Engineering, University of Science and Technology Beijing, Beijing 100083, China; libo0910@foxmail.com (B.L.); yangzhanbing@ustb.edu.cn (Z.Y.); yangfeifei361@163.com (F.Y.); liuhuasong_ustb@163.com (H.L.); tanghaiyan@metall.ustb.edu.cn (H.T.); 2 Tube & Pipe Department, Baosteel Research Institute, Baoshan Iron & Steel Co., Ltd., Shanghai 201900, China; luo_ming@baosteel.com; 3 School of Materials Science and Engineering, Shanghai University, Shanghai 200444, China

**Keywords:** C110 casing steel, semi-macro segregation induced banded structure, QT treatment, hardness

## Abstract

C110 oil well casing tubes should have high strength and corrosion resistance which is commonly used for deep wells operation containing corrosive media. In this paper, the microstructure evolution of a kind of semi-macro segregation originated banded structure in casing tubes is studied under different heat treatments. It is shown that the characteristics of the banded structure will change significantly in subsequent hot working and heat treatment processes. For the hot-rolled ones, the banded structure is composed of pearlite plus bainite. After quenching, it evolves into martensite band with high concentration solute elements. Finally, the banded structure will change into a carbide banding under the following tempering process. The temperature and cooling rate of the tempering practice show an obvious effect on the final band structure. To improve anti-SSC (sulfide stress corrosion cracking) performance, the favorable QT (quenching and tempering) practice for C110 steel should be a higher tempering temperature and a quicker cooling rate, from which the banded structure defects can be decreased together with an obvious improvement of the tube wall hardness uniformity.

## 1. Introduction

Casing and tube steels that are used in oil and gas fields with high pressure and aggressive corrosion environment require both high strength and high corrosion and hydrogen embrittlement (HE) resistance [1,2,3]. C110 grade oil casing steel is one of highest grade corrosion resistant products with a minimum yield strength requirement of 758 Mpa. During service, there is usually a popular HE failure of the steel for deep oil and gas wells operation containing wet hydrogen sulfide (H_2_S), which is known as SSC [4]. For the quality production of C110 casing tubes, the chemical composition and thermomechanical treatment processing are the main factors that can be optimized to obtain the most suitable microstructure to achieve high strength with superior SSC resistance. Sufficient additions of alloy elements such as manganese (Mn), chromium (Cr), molybdenum (Mo), nickel (Ni) and micro-alloying elements like titanium (Ti), vanadium (V) and columbium (Cb) are necessary to provide sufficient hardenability [5,6]. Appropriate concentration of alloying element additions, such as Ni, V, Mo, and Ti, are considered to have a beneficial effect on SSC resistance of steels [2]. It is generally known that SSC resistance decreases with the increase of steel strength or its variation spectrum of hardness [7]. According to previous studies, the hard phase microstructure in steel will significantly reduce the SSC resistance [1,2]. And any significant as-cast defects such as macro or semi-macro segregations will lead to a non-uniform microstructure in the final products which is detrimental to its hydrogen SSC resistance [8,9].

It has been revealed in our previous works that the segregation in the round casting for tubing during solidification will generate banded structure in the following hot rolling products. The most common form of banded structures is the pearlite/ferrite (F/P) banding which exists popularly in the most common low carbon as-rolled steels [10,11]. The formation mechanism of this kind of banded structure has been relatively clear, and there are mainly two theories. Jatczak et al. [12] believe that the formation of banded structure is closely related to the influence of alloying elements on the activity of C element in the austenite. While the others suggest that alloying elements can affect the temperature of Ar_3_ during the transformation of austenite to ferrite, which leads to the formation of banded structure [13]. However, in our earlier studies, it was found that some banded structure in hot rolled samples while produced from a kind of as-cast semi-macro segregation are easily further transformed into a kind of severe carbide banding defect even after heat treatments, in which the carbide density of the original banded regions is much higher than the normal area [14]. The enriched carbide in the matrix will become a trap of hydrogen atom and give rise to SSC failure along the carbide precipitates [15]. Presently, the high strength sour service C110 casing products must undergo quenching and tempering treatment under American Petroleum Institute (API) specification for user application [16], Therefore, it is of great significance to reveal the formation mechanism and evolution process of the harmful banded structure or defect in tempered states for the further improvement of its service property.

Previous researches on the carbide banding mainly focus on the hypereutectoid steel [17], and little attention has been paid to such carbide banding produced by tempering in the hypoeutectoid steels. The tempered carbide is mainly derived from the decomposition of the local martensite and the transformation of retained austenite. Therefore, the tempering temperature, holding time and cooling rate will affect the precipitation and formation of the carbides, which will further determine the characteristic of the carbide band structure in the tempering material.

In this paper, the carbide band structure in C110 grade high-strength and corrosion-resistant oil casing tubes is studied, and the microstructure evolution of the banded structure from its initial hot-rolled state to the quenched state and the final tempered state is traced through metallography. Besides, special attention was given to the effects of different tempering temperatures and cooling rates on the banded structure defect, and a reasonable QT treatment practice was suggested in terms of Vickers hardness and its variation through the tube wall.

## 2. Materials and Methods 

**Figure 1:**
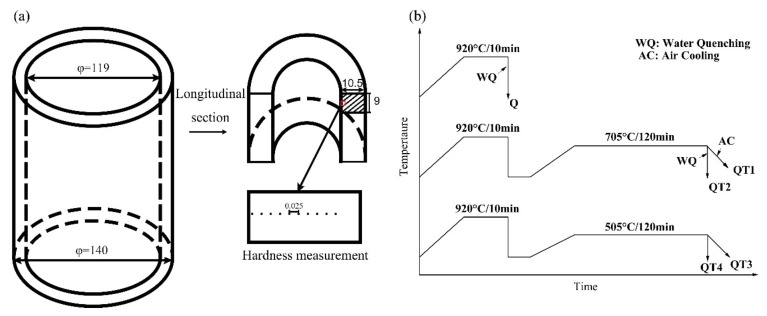
Schematic diagram of the as-rolled tube (**a**) and the specimen hot treatment processes (**b**) (unit: mm).

The C110 sample steel contains 0.2 C, 0.22 Si, 0.48Mn, 0.5Cr, 0.66Mo, and 0.1V (wt%), which is provided by Baosteel, Shanghai, China. Experimental specimens were cut from the hot rolled tube with dimension of 10.5 mm × 9 mm × 7 mm as shown in Figure 1a. Some of the as-rolled specimens were used for studying the banded structure in the hot rolling products, which is labeled as “H” in the paper. The others, labeled as “Q”, were austenitized at 920 °C for 10 min then water quenched directly based on industry practice. In the experiment, some of the as-quenched samples were tempered at 705 °C for 2 h followed by air cooling or water quenching, marked as QT1, QT2, respectively, while the other as-quenched samples were tempered at 505 °C for 2 h followed by air cooling or water quenching, which were labeled as QT3 and QT4 respectively. The applied heat treatment routes of the experimental specimens are illustrated in Figure 1b.

Steel specimens after different hot treatment processes have been ground and polished using standard metallographic preparation procedures, finishing with 1 μm diamond paste. Subsequently the microstructure was etched with 4% nital.

To reveal in detail the microstructure of different specimens, scanning electron microscopy (SEM) analysis was carried out on a JSM-6701F Field Emission Scanning Electron Microscope (JEOL, Tokyo, Japan) operating at 20 kV with the function of Energy Dispersive Spectrometer (EDS, Thermo, Ns7, Waltham, MA, USA). The segregation of alloying elements (C, Cr and Mo) has been investigated by means of Electron Probe Micro Analysis (EPMA) with a wavelength dispersive spectrometer (WDS, SHIMADZU, Wakayama, Japan). To reveal the micro-hardness changes across the banded structure region of the experimental samples, Vickers hardness measurement was applied in the high density of banded structure area, with points interval of 25 μm.

## 3. Results

### 3.1. Evolution of the Banded during the QT Hot Treatment

**Figure 2:**
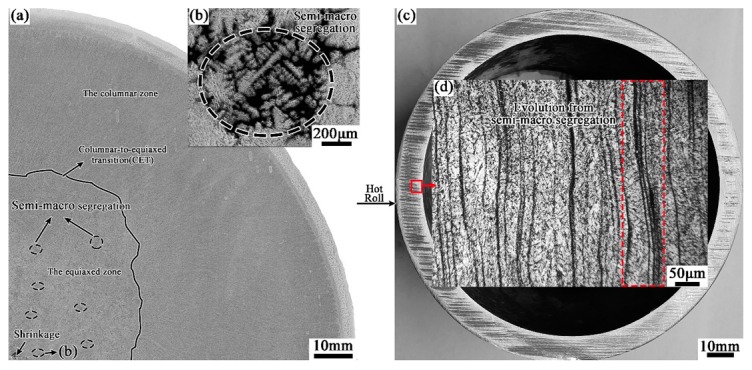
As-cast semi-macro segregation (**b**) in round casting (**a**) and its evolutionary banded defects (**d**) in tube wall (**c**).

In our previous study, the semi-macro segregation in the center equiaxed crystal zone of casting has been acclaimed as the source of the dominant banded defects in the hot-rolled tubes, which could not be effectively eliminated in the subsequent heat treatment process and would be inherited into the QT tubes, as shown in Figure 2. However, the evolution characteristic of banded structure from hot rolled state to tempered state is not clear yet, which will be explored here in-depth experimentally.

**Figure 3:**
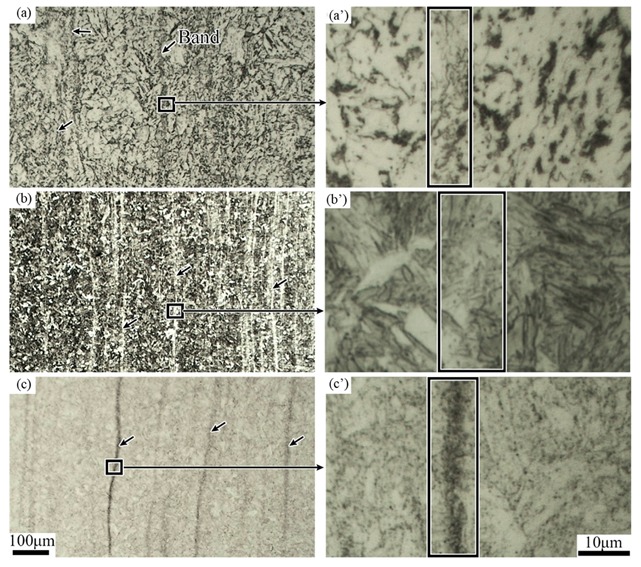
Optical micrographs of the tube banded structure for cases of (**a**) H, (**b**) Q and (**c**) QT1 specimens with their in-band higher magnification images (**a’**), (**b’**) and (**c’**), respectively.

Figure 3 shows the micrographs of the tube samples after different hot treatments, together with their typical morphology of the banded structure. The samples cooled at air after rolling are mainly composed of ferrite and pearlite, and the banded area is significantly darker as shown in Figure 3a. After quenching, in the Figure 3b, significant changes have taken place in both the banded structure (the bright region) and the matrix structure (the dark region). Figure 3b’ shows a clear lath martensite structure because of the high cooling rate. Figure 3c’ is from the tempering sample, in which it can be seen that the metallographic structure of the sample is significantly more uniform due to the high tempering temperature (705 °C) and long soaking time (2 h).

**Figure 4:**
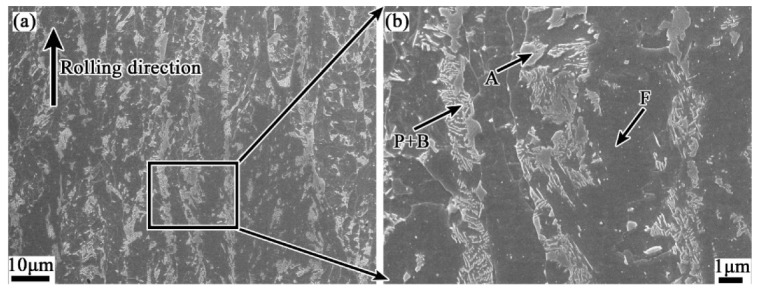
Lower (**a**) and higher (**b**) magnified scanning electron microscopy (SEM) image of banded structure in hot rolled sample. (A-austenite, B-bainite F-ferrite and P-pearlite).

**Figure 5:**
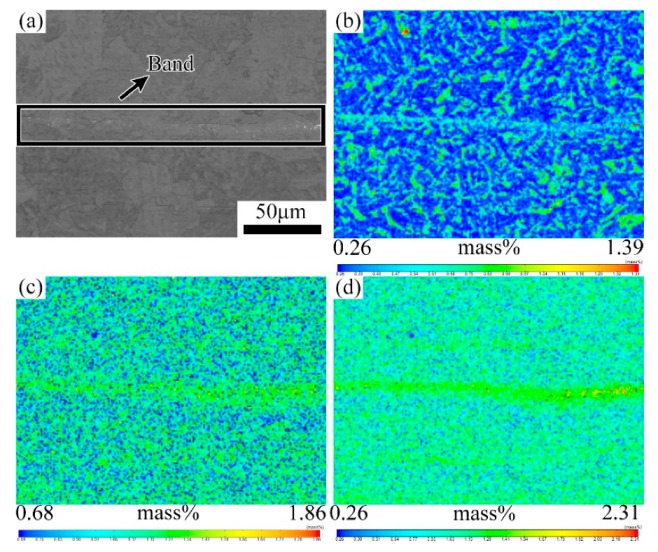
Electron probe micro analysis (EPMA) analysis of map scanning on the banded structure of hot rolled sample. (**a**) Optical micrograph of the banded structure; (**b**) EPMA maps of element C; (**c**) EPMA maps of Cr; (**d**) EPMA maps of Mo.

The microstructures of the as-rolled specimens characterized by SEM are given in Figure 4. It shows that the banded structure in the hot rolled sample is virtually discontinuous, which is mainly formed by the microstructure different from the matrix in regular arrangement along the rolling direction, as shown in Figure 4a. With higher magnifications, the banded structure is composed of pearlite, bainite and a small amount of residual austenite or martensite-austenite (M-A) islands, while the matrix area is mainly ferrite, as shown in Figure 4b. In other words, the banded structure in the hot rolled casing tube is pearlite plus bainite band. The EPMA results show that the content of solute elements in the dark banding region is clearly higher than the matrix, as shown in Figure 5.

**Figure 6:**
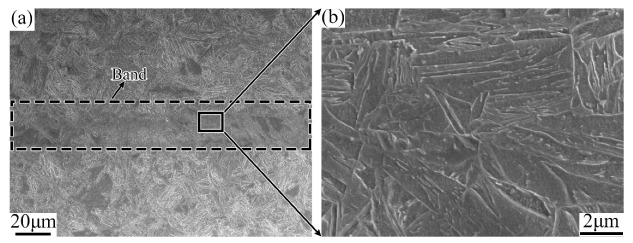
Lower (**a**) and higher (**b**) magnified SEM images of banded structure in as-quenching sample.

**Figure 7:**
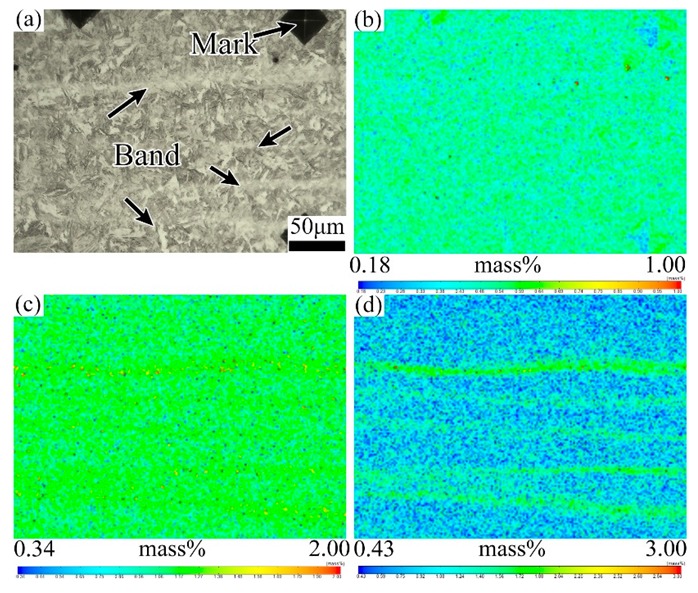
EPMA analysis of map scanning on the banded structure of as-quenching simple. (**a**) Optical micrograph of the banded structure; (**b**) EPMA maps of element C; (**c**) EPMA maps of Cr; (**d**) EPMA maps of Mo.

Figure 6 is the lower- and higher-magnified SEM photographs of the banded region in the quenched sample. It shows that there is still a significant banded microstructure after quenching. Both the banded region and the matrix region are martensitic. However, the banded region is relatively darker in color and appears as a black martensitic structure under the SEM. Further, from the results of the EPMA map scanning shown in Figure 7, it is found that the elemental distribution of the banded region changes remarkably after quenching. The concentration of the element C becomes relatively uniform as compared with the substitutional alloying elements such as Cr and Mo, which remain to be of clear banded distribution.

The enrichment of substitutional elements Cr and Mo in the banded structure region will affect the formation and growth of martensite. As shown in Figure 6a, the lamellar morphology of the martensite in the banded area is different from the normal area, which appears as a darker banded structure at the lower magnified SEM image. In other words, the banded structure in the quenching sample is martensitic with high concentration of alloy elements.

**Figure 8:**
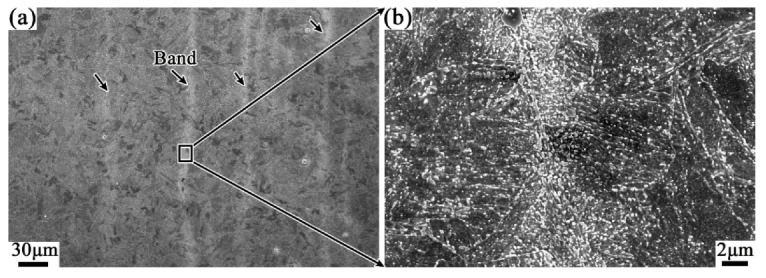
Lower (**a**) and higher (**b**) magnified SEM images of banded structure in QT1 sample.

**Figure 9:**
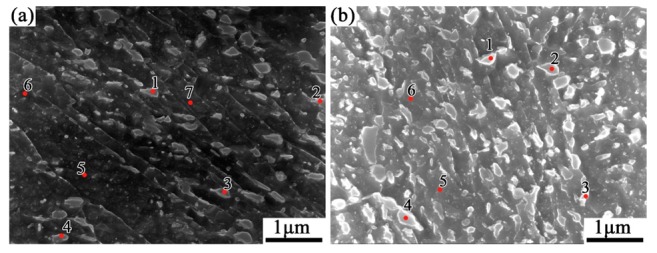
SEM images of the normal (**a**) and banded (**b**) areas of QT1 sample.

Figure 8 is the SEM image of a banded location in the QT1 sample. It can be seen that the color of the banded structure in tempering samples is brighter under the SEM, and the high magnified image shows that the banded is composed of dense particle phase. Its higher magnified SEM image is shown in Figure 9, which includes the normal area and the banded area. With consideration of the heat treatment process, it can be seen that both the normal area and the banded structure area are tempered sorbite, but the particle phase in the banded area is obviously denser.

To explore the local composition, EDS analysis is carried out on the marked location in Figure 9. The results are given in Table 1 and Table 2. In the Figure 9a, the marked positions of 1 to 4 are the particles while the points 5 to 7 are located in the normal matrix. The result shows that the C content of the particles is higher than that of the matrix, which can be identified as carbide combining with the morphology of the particle phase and the microstructure of the steel.

Table 2 shows the results of EDS analysis of the banded defect region, in which 1 to 4 points are the positions of carbide particles, and 5, 6 points locate at its matrix. Comparing the normal regions to the banded area, the latter has not only a higher C content, together with also other alloying elements, such as Ti, Cr, Mn and Nb, which are obviously originated from the semi-macrosegregation during solidification.

**Figure 10:**
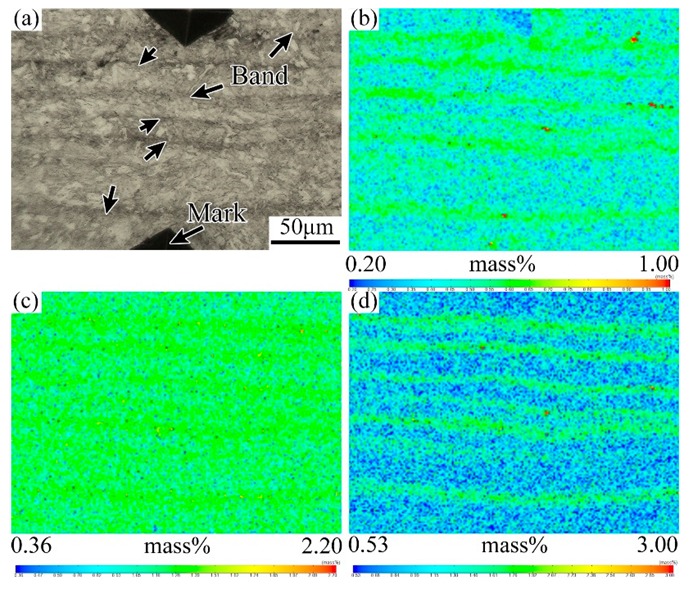
EPMA analysis of map scanning on the banded structure of QT1 sample. (**a**) Optical micrograph of the banded structure; (**b**) EPMA maps of element C; (**c**) EPMA maps of Cr; (**d**) EPMA maps of Mo.

Figure 10 demonstrates the element distribution characteristics of the banded structure region in the QT1 specimen. It can be seen that the banded structure after quenching and tempering treatment remains rich in alloy elements such as C, Cr and Mo. Combined with the SEM results, it is known that the banded structure in the tempering samples appears as dense carbide particles, which is a kind of carbide banding defect.

From the work above, it is shown that the characteristic and morphology of the banded structure will change upon different heat treatments. The forming principle and transformation process of the different banded structures will be discussed in detail later.

### 3.2. Effect of Tempering Temperature and Cooling Rate on Carbide Banding

The carbide banding in steel has been well known as an easier propagation pathway of SSC during its service process [1]. Therefore, there is a great need to control the carbide banded in the casing tube, both from the initial solidification process and from the following hot work processes. In the present work, special attention is given to the carbide precipitation period during QT treatment, i.e., the effect of soaking temperature and cooling rate on the banded structure during tempering.

**Figure 11:**
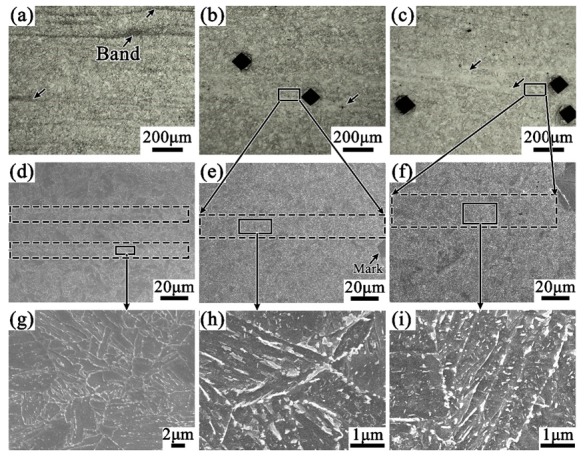
Effect of different heat treatment processes on the carbide banded. Photos downward (**a**,**d**,**g**), (**b**,**e**,**h**) and (**c**,**f**,**i**) correspond to heat treatments QT2, QT3, and QT4 in Figure 1b respectively.

It can be seen from Figure 11a that the level of banded structure is significantly reduced while the cooling pattern in the tempering process is changed from air cooling to water quenching, compared with Figure 8. Furthermore, the density of carbide particles in the banded structure is effectively reduced which can be read from the SEM images of Figure 11d,g. It suggests that increasing the cooling rate during tempering can inhibit somewhat the development of the banded structure in the casing tube.

With the same cooling pattern (air cooling), however, when the tempering temperature was lowered from 705 °C to 505 °C, the banded structure in the QT3 samples almost disappeared. Moreover, the carbide particles are obviously reduced in both the matrix and the banded region, as shown in Figure 11b,e,h. With further increasing of the cooling rate, as shown in Figure 11c,f,i, since the banding structure has been substantially eliminated due to the lower holding temperature, it is difficult to further modify the band structure by changing the cooling rates, which can be read from the minute difference in microstructure between QT3 and QT4 samples.

### 3.3. Hardness Evaluation under Different Heat Treatment Processes

**Figure 12:**
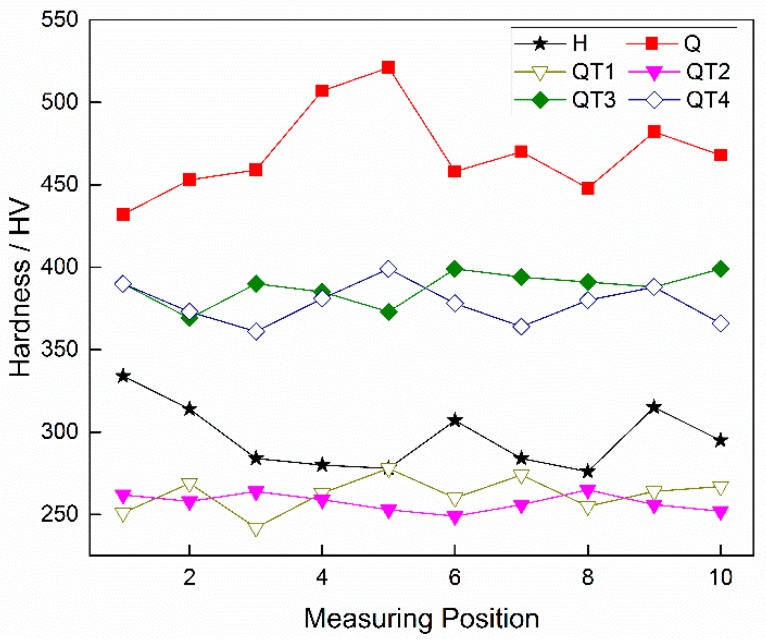
Sample hardness distribution in tube wall radial direction under different heat treatments.

In the tube inner side with dense banded structure, hardness measurement was performed in the radial direction perpendicular to the banded, and the measurement interval was 25 μm. Ten points were measured for each sample, and the results are shown in Figure 12. The measurement results of the hot rolled samples are shown by the black line. The room temperature structures in the sample are mainly ferrite and pearlite, while in the banded region there are composed of pearlite and bainite, which leads to a large difference in hardness between the banded and matrix, with maximum up to 58 HV. After quenching, the overall hardness of the sample is obviously increased due to the formation of martensite at high cooling rate, shown by the red line in the figure. In this case, there is still a severe difference in hardness in the radial direction, which can be attributed to a different phase transformation extent between the original banded structure. It has been observed that the hardness variation has been obviously improved after tempering treatments. However, the decrease in the tempering temperature results in a rise of the overall hardness value, together with a larger hardness variation, which will be harmful to the resistance of SSC [18]. In contrast, the high-temperature tempering followed by water quenching can inhibit the development of the band structure which ensure a matrix structure composed of uniform tempered sorbate. Accordingly, the hardness uniformity of the sample is much better, which should be beneficial to improve its SSC resistance performance.

## 4. Discussion

### 4.1. Evolution of Banded Structure and its Mechanism

**Figure 13:**
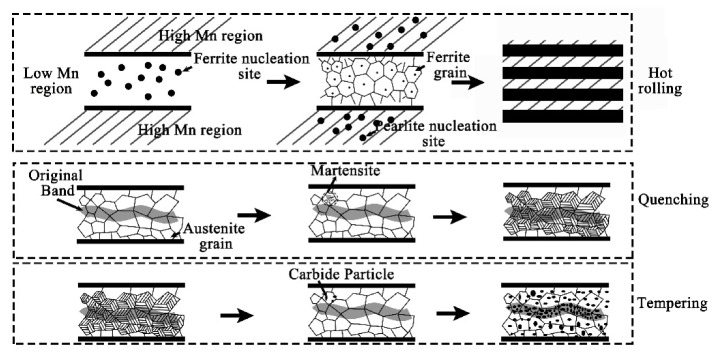
Illustration for the evolution of banded structures from hot rolling to QT treatment.

For the hot-rolled samples with banded structure, the microstructure of the region with high solute element content is pearlite plus bainite, while the poor solute region is ferrite. As is well known, there have been many studies on the formation of this type of banded microstructure. It is generally believed that alloying elements can affect the Ar_3_ transition temperature during austenite transformation. If this temperature is lowered by the solute, then proeutectoid ferrite nucleates first in the solute lean regions. Conversely, the proeutectoid ferrite forms preferentially in the solute rich regions. In this study, the enrichment of alloying elements reduces the Ar_3_ transition temperature, such as Mn as shown in the Figure 13. Therefore, the proeutectoid ferrite nucleates first in the Mn lean regions. In this case, carbon atoms, which diffuse rapidly, are rejected from the proeutectoid ferrite, thereby producing carbon rich regions of austenite, which transform eventually to pearlite and bainite.

For the following quenching process, the samples were kept at 920 °C for 10 min to austenitization. For the present sample, the soaking time of 10 min is enough to make C diffuse uniformly, while the diffusion of the substitutional alloy solutes such as Cr and Mo are very limited [19,20]. During water quenching, the high cooling rates will yield high driving force for the decomposition of austenite, and different in the Ar_3_ temperatures between the manganese-lean and manganese-rich regions should have a lesser effect [21]. Then the “P + B” banded structures will be removed and transformed into martensite. However, substitutional elements like Cr and Mo will remain in the martensite which is corresponding to the former segregation band. And the substitutional elements will also affect the martensite layer structures which results in a different morphology, a banded martensite with a higher solute content.

For the final heat treatment of high temperature tempering, it will undergo redistribution of C elements, decomposition of martensite and retained austenite, and the formation of carbides [22,23]. Due to the enrichment of the substitutional elements in the segregation banded, more carbides will be formed during tempering.

### 4.2. Effect of Tempering Temperature and Cooling Rate on the Banded Structure

The conventional banded structure in steel such as pearlite/ferrite bands has been well understood, including its formation during hot rolling or heat treatments. For the banded structure of carbides presented above, however, little attention has been given to its change or characteristic during the final tempering process. For better control of this kind of semi-macro segregation originated banded defect, the effect of tempering temperature and the following cooling rate should be discussed further from the point of view of carbides precipitation and growth during this process. Any inhibition measure to the precipitation of tempered carbides should be helpful to prevent the development of the banded defect. As we know, the tempered carbide is derived from the decomposition of martensite and retained austenite. Reducing the tempering temperature will inhibit the desolution of C element and the formation of carbides, resulting in a decrease in the amount of carbides in the banded structure. 

Similarly, rapid cooling after tempering also inhibits the formation or growth of the carbides and reduces the number of the banded defects. Luo et al. [24] revealed that the relationship of cooling rate to the carbides volume fraction can be expressed by Equation (1):

V = M × *e*^−N×Vc^, (1)

where V is the volume fraction of precipitations, pct.; Vc is the cooling rate, Ks-1; M, N are constants obtained from the nonlinear regression. Therefore, when the cooling rate is slow, the precipitates have sufficient time to nucleate and grow. As the cooling rate increases, however, the alloying elements are difficult to nucleate to form carbides, which has been also verified in our above experiments as shown in Figure 8 and Figure 11. Additionally, the density of the banded carbides decreases together with the increasing cooling rate.

## 5. Conclusions

The evolution of the semi-macro segregation originated banded structure under different heat treatment processes has been studied experimentally for C110-grade high strength corrosion resistant casing steel. The main conclusions are as follows:

- The banded structure originated from as-cast semi-macro segregation will change significantly in subsequent hot working and heat treatment processes. The hot rolled state of the banded structure is mainly composed of pearlite, together with few bainite. After quenching during QT process, the C element diffuses obviously, but the morphology of the banded defect structure changes little. The microstructure, however, has been transformed into martensite with high concentration of alloy elements. After the following tempering treatment, the banded martensite structure will evolve into a kind of banded defect full of dense particles of various carbides.
- Compared with the conventional heat treatment process of these steel products, a lower tempering temperature and a quicker cooling rate after tempering can reduce the amount and density of carbide precipitation in the banded structure to a certain extent, thereby reducing the degree of the banded defects. However, the radial microstructure hardness test of the casing tube wall shows that, compared with a tempering temperature of 705 °C, both the overall hardness and the hardness variation of the sample are larger under 505 °C, which cannot meet the requirements of anti-SSC performance; however, under the condition of tempering at 705 °C and a following water cooling, instead of the conventional air cooling process, the banded structure defects can be decreased together with an obvious improvement of hardness uniformity.

## Figures and Tables

**Table 1:** Energy Dispersive Spectrometer (EDS) analysis of the tube normal microstructure in Figure 9a (mass fraction/%).

Position	C	Fe
1	1.63	98.37
2	1.71	98.29
3	1.90	98.10
4	1.82	98.18
5	1.23	98.77
6	1.37	98.63
7	1.16	98.84

**Table 2:** EDS analysis of tube banded area in Figure 9b (mass fraction/%).

Position	C	Ti	Cr	Mn	Fe	Nb	Mo
1	2.34	6.73	-	-	85.44	5.49	-
2	2.59	-	0.83	1.68	94.90	-	-
3	2.11	-	1.38	-	96.51	-	-
4	2.46	-	-	-	97.54	-	-
5	1.39	-	-	-	96.53	-	2.09
6	1.11	-	-	-	98.89	-	-
